# Monitoring Molecular Residual Disease in Colorectal Cancer Using Tumor-Informed ctDNA Analysis

**DOI:** 10.32604/or.2026.080218

**Published:** 2026-10-28

**Authors:** William C. Cho, Yingyu Wang, Qianqian Yao, Tam Berntsen, George Yeung, Paul Tang, Tobias Wittkop, Li Weng, Lui Ng, Dominic C. C. Foo

**Affiliations:** 1 Department of Clinical Oncology, Queen Elizabeth Hospital, Hong Kong SAR, China; 2 Department of Research and Development, AccuraGen Inc., San Jose, CA, USA; 3 Department of Surgery, University of Hong Kong, Hong Kong SAR, China

**Keywords:** Circulating tumor DNA, molecular residual disease, colorectal cancer, liquid biopsy, recurrence prediction

## Abstract

**Background:** The early detection of molecular residual disease (MRD) is critical for predicting recurrence and guiding management in colorectal cancer (CRC). We aimed to evaluate the performance of tumor-informed circulating tumor DNA (ctDNA) analysis in monitoring MRD after curative-intent surgery. **Methods:** In this cohort study of 28 resected CRC patients, tumor-informed variants were identified from formalin-fixed paraffin-embedded (FFPE) or fresh-frozen (FF) tissues using whole-genome sequencing. Post-operative plasma ctDNA was analyzed with the next-generation sequencing-based AccuScan platform at landmark (2–6 weeks) and longitudinal time points. **Results:** ctDNA-based MRD detection achieved 100% specificity (95% CI: 78.2–100%) and positive predictive value for recurrence in this cohort of 28 patients, which included 13 recurrence events with a median follow-up of 36.2 months. Sensitivity was 83.3% (95% CI: 35.9–99.6%) for patients with FFPE-guided analysis (*n* = 13) and 85.7% (95% CI: 42.1–99.6%) for the combined cohort (FFPE- and/or FF-guided analysis (*n* = 20) at the landmark time point, improving to 88.9% and 92.3%, respectively, with longitudinal monitoring. MRD-positive status was associated with significantly reduced disease-free survival (*p* < 0.0001) in unadjusted analyses. Although FFPE-derived sequencing introduced more artifacts, bioinformatic filtering preserved assay accuracy, and the concurrent use of both reference types in a subset of patients highlighted the potential to improve overall detection sensitivity. **Conclusions:** These findings suggest that tumor-informed ctDNA analysis provides a promising approach for post-operative MRD surveillance and recurrence risk stratification in CRC. However, the small sample size, lack of external validation, and absence of neoadjuvant-treated patients preclude definitive conclusions. Independent prospective validation is required.

## 1 Introduction

Colorectal cancer (CRC) remains a major global health burden, and patient outcomes are heavily influenced by the early detection of residual disease and timely clinical intervention [1]. Although surgical techniques and systemic therapies have advanced, recurrence rates remain substantial, highlighting the urgent need for highly sensitive biomarkers to detect molecular residual disease (MRD) early and guide treatment decisions [2].

Post-operative (post-op) surveillance currently depends on periodic imaging and serum markers like carcinoembryonic antigen (CEA), but these methods are limited in their ability to detect early relapse or precisely define recurrence risk. This diagnostic shortfall has created a pressing need for more sensitive and specific biomarkers of MRD. In recent years, circulating tumor DNA (ctDNA) has emerged as a powerful tool for MRD assessment, providing real-time monitoring of tumor dynamics and recurrence risk [3,4]. Key clinical trials (including the DYNAMIC trial in stage II colon cancer and the CIRCULATE-Japan study in resectable CRC) have demonstrated the clinical utility of ctDNA for guiding adjuvant therapy and predicting recurrence [5,6]. Tumor-informed ctDNA assays, which leverage patient-specific mutations identified from tumor tissue, enhance detection sensitivity and specificity, offering a tailored approach to recurrence surveillance [7].

Among ctDNA detection strategies, the tumor-informed approach represents a significant leap forward. By first performing deep sequencing of the resected tumor to define a patient-specific mutation profile, this method creates a personalized genetic fingerprint. Subsequent plasma samples are then screened for these exact variants, greatly increasing both sensitivity and specificity for MRD detection over non-targeted assays. The AccuScan platform uses rolling circle amplification (RCA) combined with concatemer error correction to achieve an ultra-low error rate, enabling highly sensitive detection of ctDNA [8,9,10,11]. However, the success of this approach depends heavily on the quality and accessibility of the tumor tissue used for profiling. In clinical practice, formalin-fixed, paraffin-embedded (FFPE) blocks are the most readily available tissue source, while fresh-frozen (FF) specimens (though considered optimal for molecular analysis) pose greater logistical challenges for collection, storage, and handling. It is widely recognized that FFPE processing can induce DNA damage and sequencing artifacts, raising concerns about potential false-positive variant calls [12,13]. Whether these technical limitations meaningfully compromise the clinical utility of FFPE-informed ctDNA assays, and how their performance stacks up against assays guided by FF tissue, are questions that remain open.

This study aimed to assess the assay’s performance in predicting disease recurrence (including its sensitivity, specificity, and predictive values), using routinely available FFPE tumor tissue against FF tissue as the reference for identifying patient-specific tumor variants, and determine the prognostic value of post-op ctDNA status by examining its association with disease-free survival (DFS). We hypothesized that a tumor-informed ctDNA approach would offer a reliable and sensitive method for post-op surveillance, and that acquiring a set of tumor-specific variants from FFPE samples is non-inferior to that from FF tissue for the accuracy of recurrence prediction.

## 2 Materials and Methods

### 2.1 Study Design and Patient Cohort

The patient cohort was collected at Queen Elizabeth Hospital (Hong Kong SAR, China) and the University of Hong Kong (Hong Kong SAR, China) from January 2018 to December 2021. Eligible patients met the following criteria: (1) Histologically confirmed CRC, (2) Completion of curative-intent surgical resection, (3) Availability of either FFPE or FF tumor tissue, (4) Availability of post-op plasma samples, and (5) Event-free patients were followed for a minimum of two years. The exclusion criteria for the study cohort were: (1) Insufficient cell-free DNA (cfDNA) yield (<5 ng), (2) Significant genomic DNA (gDNA) contamination of cfDNA, (3) Inadequate tumor tissue, (4) Lost to follow-up. The study protocol was approved by the Institutional Review Board of the University of Hong Kong/Hospital Authority Hong Kong West Cluster (IRB Reference: UW 23-495) and conducted in accordance with the Declaration of Helsinki. Written informed consent was obtained from all participants. From an initial cohort of 36 patients, eight were excluded due to insufficient cfDNA yield (<5 ng, *n* = 2), significant gDNA contamination of cfDNA (*n* = 1), inadequate tumor tissue (*n* = 3), or lost to follow-up (*n* = 2). The final analysis included 28 patients with complete clinicopathological and molecular data.

### 2.2 Sample Processing and Quality Control

Tumor specimens were processed immediately upon collection. FF tissues were snap-frozen in liquid nitrogen and stored at −80°C. FFPE samples were prepared by fixing tissues in 10% neutral buffered formalin for 24 to 48 h, followed by routine dehydration and paraffin embedding. For histologic review, 5-μm sections were cut from FFPE blocks using a Shandon Finesse 325 microtome (Thermo Fisher Scientific, Waltham, MA, USA), and from FF tissues using a Leica CM 1860 UV cryostat (Leica Biosystems, Nussloch, Germany). For FFPE sections, slides were first deparaffinized in xylene (Catalog No. 66-15, Epredia, Kalamazoo, MI, USA) and rehydrated through a graded ethanol series (Catalog No. E/0650DF/C17, Thermo Fisher Scientific). For FF sections, slides were briefly fixed in cold acetone and rinsed. Subsequently, both types of sections were stained in Gill’s hematoxylin solution II (Catalog No. GHS216, Sigma-Aldrich, St. Louis, MO, USA) for 3–5 min, rinsed in running tap water for 5 min, differentiated in 1% acid alcohol for 10–20 s, and blued in Scott’s tap water substituted (Catalog No. S5134, Sigma-Aldrich) for 1–2 min. Sections were stained with Eosin Y (Catalog No. 6766010, Epredia) for 20 s and subsequently dehydrated through 95% ethanol and absolute ethanol. The tissue sections were mounted with DPX mountant (Catalog No. 06522, Sigma-Aldrich) [14]. Stained slides were independently evaluated by two board-certified pathologists under an Olympus DP71 microscope (Olympus Corporation, Tokyo, Japan). Only samples with a tumor cellularity of at least 20% were used for downstream extraction.

### 2.3 DNA Extraction and Quantification

gDNA from tumor tissue and peripheral blood mononuclear cells, along with cfDNA, were extracted following the manufacturers’ recommended protocols. gDNA was isolated from five FFPE sections (5-μm thickness) using the High Pure FFPET DNA Isolation Kit (Catalog No. 06650767001, Roche, Basel, Switzerland), with elution in 25 μL of elution buffer, or 30–60 mg of FF tissue. Plasma volumes ranging from 1 to 3 mL were processed using the MiniMax High Efficiency Cell-Free DNA Isolation Kit (Catalog No. A17622CN-384, Apostle Bio, Pleasanton, CA, USA), with elution in 80 μL of Tris-EDTA buffer. gDNA concentrations were measured by NanoDrop spectrophotometer (Catalog No. ND-ONE-W, Thermo Fisher Scientific), while cfDNA yield was quantified using Qubit 3.0 Fluorometer (Catalog No. Q33216, Thermo Fisher Scientific). DNA integrity was assessed with Agilent 2100 Bioanalyzer (Catalog No. G2939BA, Agilent Technologies, Santa Clara, CA, USA).

### 2.4 Whole-Genome Sequencing (WGS) for Tumor-Specific Variant Detection

gDNA was sheared to ~300 bp fragments using the M220 Ultrasonicator (Catalog No. 500295, Covaris, Woburn, MA, USA). Libraries were prepared using 100 ng of fragmented DNA input with the KAPA Hyper Prep Kit (Catalog No. KK8504, Roche, Basel, Switzerland), following the manufacturer’s protocol. Paired-end 150 bp sequencing was performed on the DNBSEQ-T7 platform (MGI Tech, Shenzhen, China; https://en.mgi-tech.com/products/instruments_info/22/), an ultra-high-throughput sequencing system based on DNBSEQ™ technology. The average sequencing depth for tumor samples was 42.45×. Sequencing reads were demultiplexed and processed with Sentieon^®^ TNseq^®^ (version 202112.02) for variant calling using a matched normal (peripheral blood mononuclear cell) sample, as previously described [11]. Candidate variants were subsequently refined through downstream filtering to retain high-confidence tumor-informed markers for plasma analysis. To reduce false positives, we excluded probable germline variants present in the gnomAD database (v3.1.2; https://gnomad.broadinstitute.org) with a population allele frequency > 0.0001 and clonal hematopoiesis mutations (by reviewing variants present in the peripheral blood mononuclear cell sample at low VAF). This conservative threshold was selected based on internal validation analyses to minimize the contribution of rare germline and recurrent background variants that may affect specificity in ultra-sensitive MRD detection. This study focused on single-nucleotide variants for MRD tracking; insertions/deletions, structural variants and copy number alterations were not analyzed. Variants were annotated using ANNOVAR (version 2020-06-08; http://annovar.openbioinformatics.org) with reference to the COSMIC (v97; https://cancer.sanger.ac.uk/cosmic), ClinVar (20220320; https://www.ncbi.nlm.nih.gov/clinvar/), and dbSNP (v155; https://www.ncbi.nlm.nih.gov/snp) databases [15]. Additional FFPE-specific artifact filtering included orientation bias filters and exclusion of variants in low-complexity regions.

### 2.5 AccuScan Platform for MRD Detection in Plasma Samples

cfDNA libraries were prepared following established protocols [8,9,10,11]. In brief, cfDNA was heat-denatured to produce single-strand DNA, which was then circularized through intramolecular ligation using a single-strand DNA ligase capable of ligating DNA fragments at least 15 bases in length [8]. The circularized templates underwent whole-genome rolling circle amplification (RCA) using phi29 DNA polymerase (New England Biolabs, Ipswich, MA, USA), initiated with random hexamer primers. The reaction was performed under isothermal conditions in accordance with manufacturer recommendations and previously described concatemer-based sequencing approaches [9,11]. The resulting RCA products were subsequently fragmented via sonication to an average size of approximately 500 base pairs. Library preparation from the fragmented RCA products was carried out using the NEBNext^®^ Ultra™ II Ligation Module (Catalog No. E7595L, New England Biolab, Ipswich, MA, USA), along with the NEBNext^®^ Ultra™ II End Repair/dA-Tailing Module (Catalog No. E7546L, New England Biolab, Ipswich, MA, USA). Final libraries were sequenced on the DNBSEQ-T7 platform (Complete Genomics; a subsidiary of MGI Tech, Shenzhen, China; https://en.mgi-tech.com/products/instruments_info/22/) using the DNBSEQ-T7RS High-throughput Sequencing Kit (FCL PE150; MGI Tech, Shenzhen, China).

Variant calling based on concatemer analysis was performed as previously described [8,11]. Briefly, paired-end FASTQ files were processed through a custom pipeline deployed on Amazon Web Services cloud infrastructure [16]. This pipeline generated variant calls, molecule-level depth estimates across the genome, and sample-specific error profiles. Initial read alignment to the human reference genome (hg38) was conducted using bwa mem [17]. Reads arising from the same original cfDNA molecule were grouped by analyzing the proximity and alignment coordinates of paired-end reads to infer ‘molecule boundaries’, enabling accurate molecular coverage estimation. Molecule-level depth was computed at each genomic position for both variants, with reference base calls requiring repeat confirmation. Only high-quality bases were included in the analysis. A base was considered “repeat-confirmed” if it was called consistently across the two tandem copies (the concatemerized repeats as a result from the rolling circle amplification of the original circularized molecule). Sequence deviations from the reference that met this criterion were classified as true variants, while inconsistencies between tandem copies were attributed to random sequencing errors and excluded. Repeat confirmed reference bases contributed to the final molecular depth calculations. The AccuScan MRD algorithm was applied to plasma samples using the error-corrected variant calls generated through this pipeline as previously described [11]. To prevent artifacts from compromising specificity, we first filtered the personalized tumor variant list prior to plasma analysis, excluding loci known to be prone to background noise based on prior validation experiments (e.g., variants occasionally observed as single-molecule events in negative controls, as well as C>T transitions at CpG sites associated with FFPE-induced artifacts). The molecule-level decision framework used in this study was empirically derived from internal validation experiments in the ultra-low ctDNA setting. During plasma analysis, each tumor-informed variant was evaluated at the molecule level. In the ultra-low tumor-fraction setting typical of post-operative samples, true ctDNA signal is frequently observed as sparse single-molecule events distributed across multiple independent tumor-informed loci. During assay development and validation, recurrent multi-molecule observations at specific loci were empirically enriched in negative-control plasma samples and were therefore considered likely background-prone or artifactual events. Accordingly, variants supported by exactly one unique molecule were retained as informative, while variants supported by two or more molecules were excluded. This threshold was empirically determined to maximize specificity while preserving sensitivity in the post-op window. The circulating variant allele fraction (cVAF) was calculated as an aggregate across all variants evaluated for the patient. Plasma MRD positivity was determined using a statistical framework as previously described [11], which integrates signals across the full set of tumor-informed variants and defines positivity by rejection of the null hypothesis (no tumor DNA present) under a predefined specificity threshold.

### 2.6 Statistical Analysis

Tumor-specific variants were identified and analyzed according to cancer stage and recurrence status. Longitudinal tracking of cVAF was performed to monitor molecular dynamics over time. DFS was defined as the interval between surgical resection and either documented disease recurrence or the last follow-up for censored patients. DFS was assessed using Kaplan-Meier survival analysis with log-rank test at both landmark (2–6 weeks post-surgery) and longitudinal time points. For comparisons between two independent groups, we used the Wilcoxon rank-sum test. Paired comparisons (such as between matched FFPE and FF samples) were evaluated with the Wilcoxon signed-rank test. Linear regression was used to assess correlations between continuous variables. All statistical analyses and data visualizations were conducted using R software (version 4.4.3; R Foundation for Statistical Computing, Vienna, Austria). A two-sided *p*-value < 0.05 was considered statistically significant.

## 3 Results

### 3.1 Patient Characteristics

A total of 28 patients met the eligibility criteria and were included in the final analysis (Fig. 1). Demographic and clinical characteristics are summarized in Table 1. The cohort had a mean age of 64 ± 10 years, with a predominance of male patients (61%, *n* = 17). All patients underwent R0 resection (curative-intent surgery with no microscopic residual tumor). Tumor staging based on the American Joint Committee on Cancer/Union for International Cancer Control 8th edition included stage I (4%, *n* = 1), stage II (25%, *n* = 7), stage III (54%, *n* = 15), and stage IV (18%, *n* = 5). Most tumors (79%) originated in the left colon, particularly in the rectum (43%) and sigmoid colon (25%). Histopathological analysis showed that 93% of tumors were moderately differentiated. None of the patients received neoadjuvant therapy, while 75% (*n* = 21) underwent adjuvant treatment (Table 1, Supplementary Table S1).

**Figure 1:**
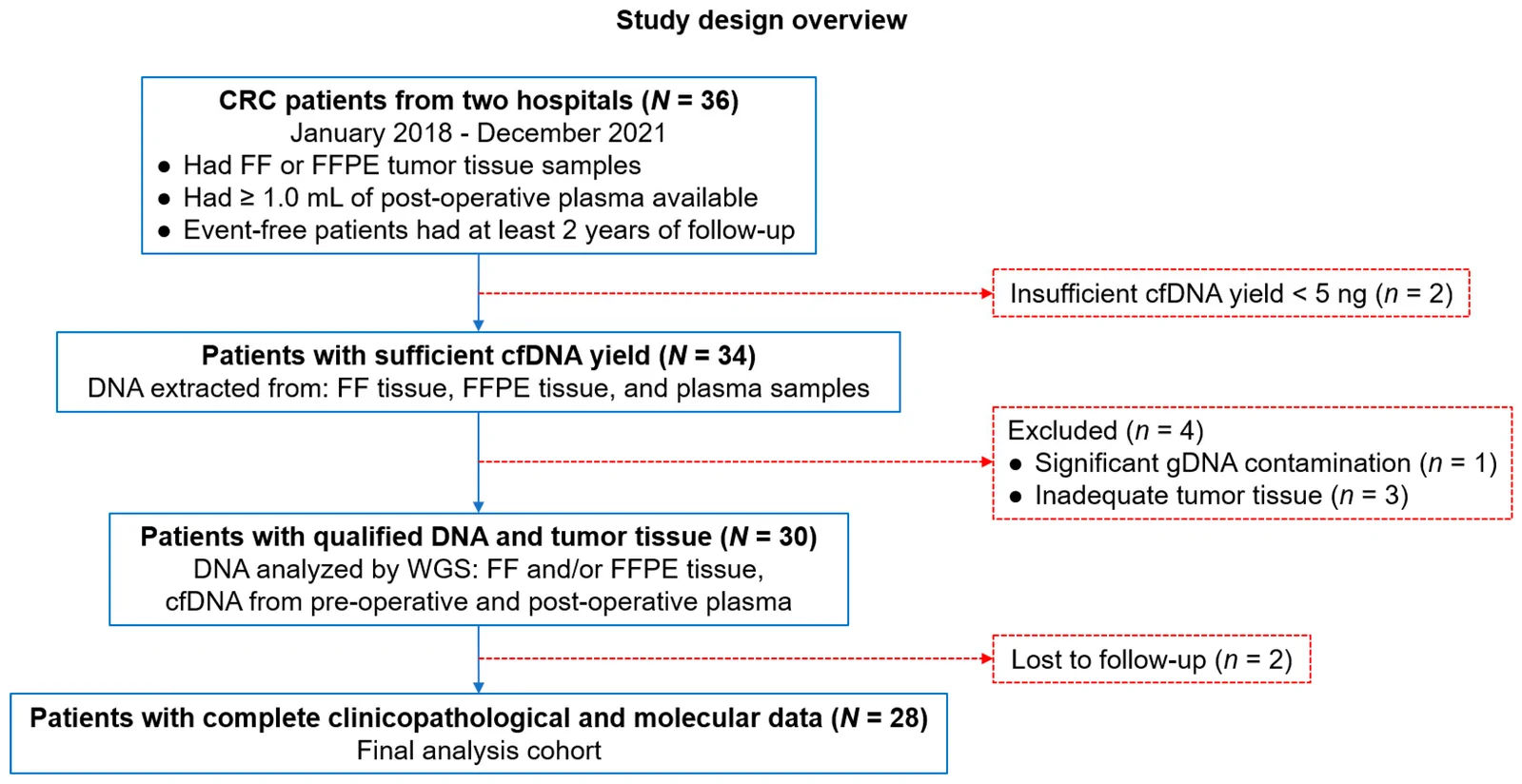
**Schematic diagram illustrating the experimental workflow, including patient enrollment, sample collection, and molecular analysis procedures.** CRC, colorectal cancer; *N*, total number of excluded patients; *n*, number of samples; FFPE, formalin-fixed paraffin-embedded; FF, fresh-frozen; cfDNA, cell-free DNA; gDNA, genomic DNA; WGS, whole genome sequencing.

**Table 1:** Demographic and clinical characteristics of colorectal cancer patients (*n* = 28).

Variables	Number, *n*	Percentage (%)
Gender		
Male	17	61
Female	11	39
Age (years)		
Mean [range]	64 [37–86]	
Tumor location		
Left	22	79
Right	6	21
Tumor site		
Rectum	12	43
Sigmoid	7	25
Colon	7	25
Caecum	2	7
Tumor differentiation		
Well	2	7
Moderately	26	93
TNM classification (tumor)		
T_2_	4	14
T_3_	18	64
T_4_	6	21
TNM classification (node)		
N_0_	8	29
N_1_	16	57
N_2_	4	14
Cancer staging		
I	1	4
II	7	25
III	15	54
IV	5	18
Adjuvant treatment		
No adjuvant treatment	7	25
Chemotherapy	20	71
Chemotherapy and targeted therapy	1	4

### 3.2 Tumor-Informed Variant Detection

WGS identified tumor-specific variants in both FFPE and FF tissue samples. Tumor samples were sequenced to an average depth of 42.45× (range, 23.75×–65.85×). Analyzable data were available for 18 FFPE and 16 FF samples, including six matched FFPE-FF pairs. A summary of tumor-specific variant counts detected across all samples is provided in Supplementary Tables S2 and S3. All tissue samples carried thousands of somatic variants. In paired samples, a median of 2595 [interquartile range (IQR): 2417–3689] genomic alterations were detected across both FFPE and FF tissues, including a median of 4844 (IQR: 4159–4952) variants unique to FFPE samples and a median of 998 (IQR: 552–2760) variants exclusive to FF tissues. Because the shared, unique, and total variant counts were summarized independently as medians across the six matched pairs, the reported median values are not expected to be additive. A median of 72.6% of variants were consistently detected across both sample types, supporting the reliability of tumor-informed profiling for ctDNA assay design.

In unpaired analysis, FFPE samples exhibited a significantly higher median variant burden (10,277 variants; IQR: 7268–20,038) than FF tissues (5771 variants; IQR: 4206–6935; *p* = 0.003), Wilcoxon rank-sum test (Fig. 2a). However, the median VAF was lower in FFPE tissues (14.69%; IQR: 12.94–20.38%) than in FF samples (21.38%; IQR: 19.11–28.23%; *p* = 0.059), Wilcoxon rank-sum test (Fig. 2b). In the six matched pairs (*n* = 6), a higher variant count was observed in FFPE samples (median: 8191; IQR: 7026–8779) than FF tissues (median: 4261; IQR: 3374–7398; *p* = 0.031), Wilcoxon rank-sum test (Fig. 2c), with lines connecting the paired samples, while the difference in VAF between paired samples was not statistically significant (*p* = 0.156), Wilcoxon signed-rank test (Fig. 2d). Given the small sample size (*n* = 6) of matched pairs, these comparisons should be considered exploratory only. Examining the VAF distribution in matched samples (Supplementary Fig. S1) revealed that shared mutations tended to have higher VAFs, whereas mutations unique to FFPE were enriched at lower frequencies (a pattern consistent with artifactual variants).

**Figure 2:**
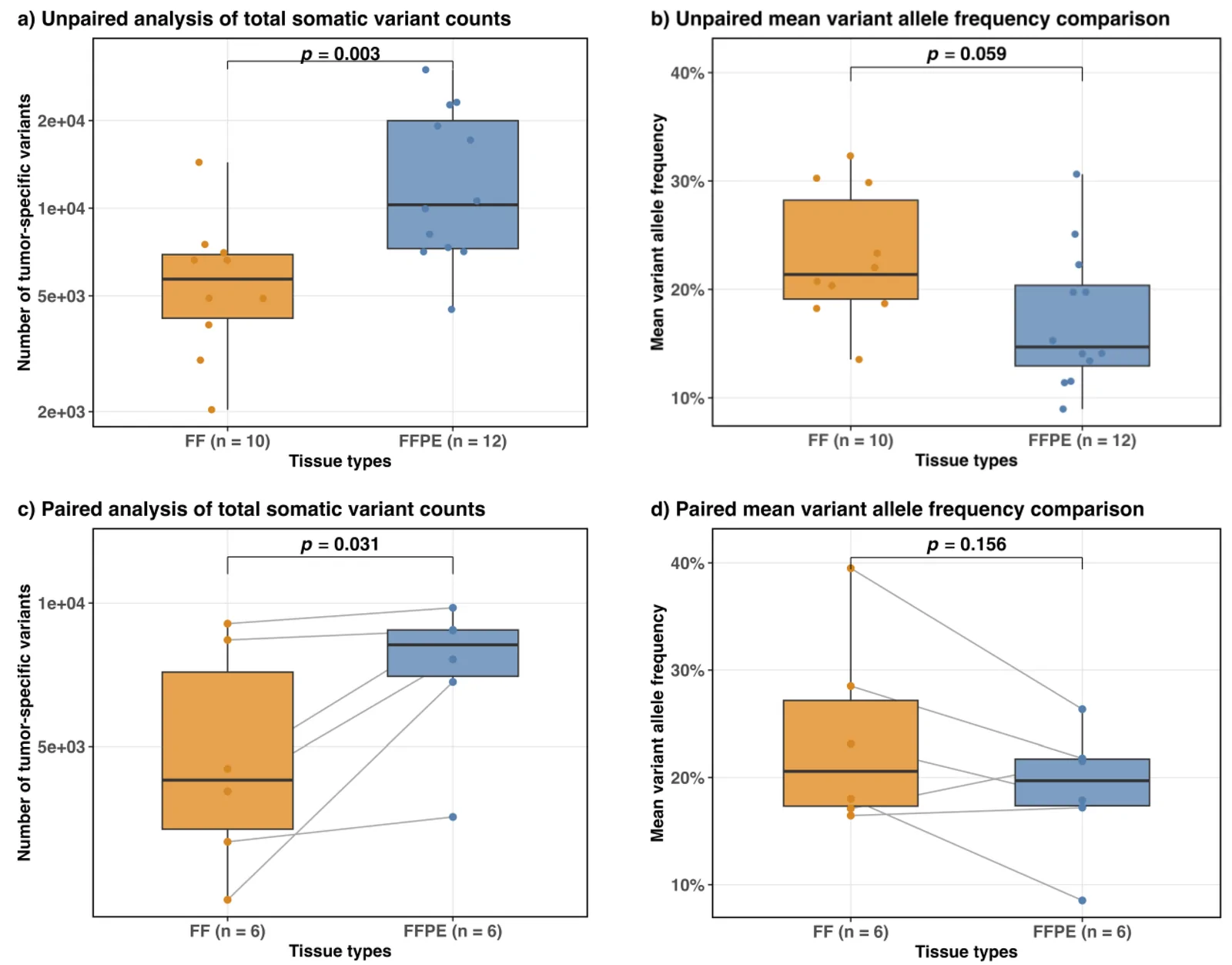
**Mutational profile comparison between FF and FFPE tumor samples.** (**a**) Unpaired analysis of total somatic variant counts in FF (*n* = 10) versus FFPE samples (*n* = 12). (**b**) Unpaired mean VAF comparison between FF (*n* = 10) and FFPE specimens (*n* = 12). (**c**) Paired analysis of somatic variant counts in matched FFPE-FF samples (*n* = 6). (**d**) Paired mean VAF comparison between matched FFPE and FF specimens (*n* = 6). FF, fresh-frozen; FFPE, formalin-fixed paraffin-embedded; VAF, variant allele frequency. Panels **a**,**b**: Wilcoxon rank-sum test; Panels **c**,**d**: Wilcoxon signed-rank test.

Mutational signature analysis of matched samples revealed a significant enrichment of A>T transversions in FFPE tissues (*p* = 0.031), Wilcoxon signed-rank test, while the frequencies of other base substitutions (A>C, A>G, C>A, C>G, C>T) were comparable between preservation methods (*p* > 0.05 for all) (Fig. S2). However, because our study did not systematically collect or control for institutional tissue processing variables (e.g., formalin fixation time, pH, or temperature), we cannot exclude the possibility that the observed A>T enrichment reflects site-specific processing conditions rather than a generalizable FFPE-associated artifact. This uncertainty limits the generalizability of our mutational signature findings. Unlike some previous studies, we did not observe significant C>T enrichment in our matched FFPE samples, which likely reflects the effectiveness of our bioinformatic pipeline in filtering out formalin-induced deamination artifacts (e.g., excluding variants in low-complexity regions and applying orientation bias filters). However, we cannot exclude the possibility that institutional differences in tissue processing (e.g., fixation time or formalin pH) may have contributed to the observed A>T enrichment. Supplementary Fig. S3 shows a stacked bar plot of the fractional contribution of each substitution type for all 28 tumor samples, illustrating the overall similarity in mutational spectra across sample types. Supplementary Fig. S4 shows the distribution of VAFs for the variants selected from tissue for tracking in plasma. The selected variants are predominantly clonal (high VAF), as expected for optimal MRD monitoring.

### 3.3 Pre-Operative (Pre-Op) and Post-Operative (Post-Op) ctDNA Detection

We performed MRD detection using AccuScan on pre- and post-op plasma cfDNA samples. As detailed in Supplementary Table S4, ctDNA variants were detectable in all 16 patients’ pre-op plasma samples. No significant differences in pre-op cVAFs were observed between FFPE- and FF-derived standards (*p* > 0.05). Additionally, tumor stages and recurrence status showed no association with pre-op cVAF. The cVAF of pre-op cfDNA showed no correlation with the mean VAF of tissue samples and DFS. The post-op landmark cVAF distribution by tumor stage was also shown (Supplementary Fig. S5). Most tracked variants were already detectable in pre-op plasma (Supplementary Table S5). Variants appearing only after surgery were rare.

### 3.4 Post-Op ctDNA-Based MRD Predicts Recurrence

Swimmer plots (Fig. 3) and performance metrics (Table 2) demonstrated 100% specificity for recurrence prediction across all assessments. However, the confidence intervals (CIs) for sensitivity estimates are extremely wide (e.g., 35.9–99.6% for FFPE landmark). Sensitivity, however, varied by sample type and timing. At the landmark time point (2–6 weeks post-surgery), sensitivities of ctDNA-based MRD detection were 83.3% (5/6 recurrences detected; 95% CI: 35.9–99.6%) for FFPE-derived samples, 100% (1/1; 95% CI: 2.5–100%) for FF-derived analyses, and 85.7% (6/7; 95% CI: 42.1–99.6%) when a positive result from either source (combined) was considered. During longitudinal monitoring, sensitivities were 88.9% (8/9; 95% CI: 51.8–99.7%), 71.4% (5/7; 95% CI: 29.0–96.3%), and 92.3% (12/13; 95% CI: 64.0–99.8%), respectively. Both tissue types achieved a perfect (100%) positive predictive value (PPV), with the number of true positives and false positives detailed in Supplementary Table S6. Negative predictive value (NPV) was slightly lower in landmark assessments (FFPE: 87.5%; combined: 92.9%) compared to longitudinal monitoring (FFPE: 90%; combined: 93.8%). Similarly, the accuracy followed the same trend as NPV, with 92.3% for FFPE, 95.0% for combined in landmark assessment, compared to 94.4% for FFPE, 96.4% for combined in longitudinal monitoring. It is important to note that the seemingly perfect performance of the FF-derived assay may be attributed to the limited sample size (Supplementary Table S6).

**Figure 3:**
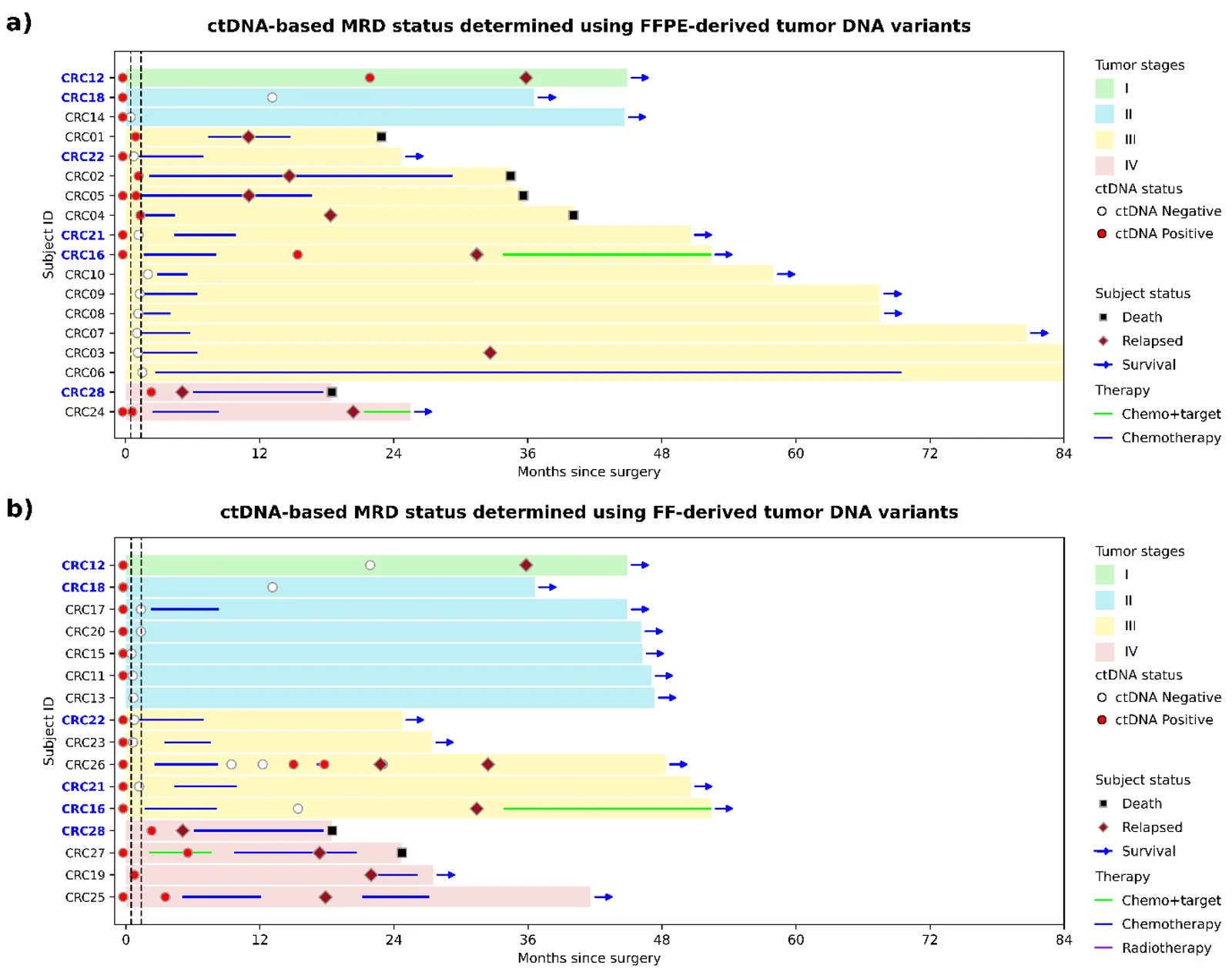
**ctDNA-based MRD status predicts colorectal cancer recurrence.** (**a**) Swimmer plot of recurrence outcomes with ctDNA-based MRD status determined using FFPE-derived tumor DNA variants. (**b**) Corresponding analysis using FF-derived tumor DNA variants. The two dashed vertical lines mark the landmark time window (2–6 weeks after surgery). Subject IDs for patients with paired FFPE and FF samples are highlighted in bold blue. ctDNA, circulating tumor DNA; MRD, molecular residual disease.

**Table 2:** Performance of ctDNA-based MRD detection in recurrence prediction using FFPE and FF tissue samples.

Time Points	Metrics	FFPE	FF	Combined
Longitudinal	Total patients, *N*	18	16	28*
	Sensitivity, % (95% CI) [*n*/*N*]	88.9 (51.8–99.7) [8/9]	71.4 (29.0–96.3) [5/7]	92.3 (64.0–99.8) [12/13]
	Specificity, % (95% CI) [*n*/*N*]	100 (66.4–100) [9/9]	100 (66.4–100) [9/9]	100 (78.2–100) [15/15]
	PPV, % (95% CI) [*n*/*N*]	100 (63.1–100) [8/8]	100 (47.8–100) [5/5]	100 (73.5–100) [12/12]
	NPV, % (95% CI) [*n*/*N*]	90 (58.7–98.3) [9/10]	81.8 (58.2–93.6) [9/11]	93.8 (69.5–99) [15/16]
	Accuracy, % (95% CI) [*n*/*N*]	94.4 (72.7–99.9) [17/18]	87.5 (61.7–98.5) [14/16]	96.4 (81.6–99.9) [27/28]
Landmark	Total patients, *N*	13	9	20^#^
	Sensitivity, % (95% CI) [*n*/*N*]	83.3 (35.9–99.6) [5/6]	100 (2.5–100) [1/1]	85.7 (42.1–99.6) [6/7]
	Specificity, % (95% CI) [*n*/*N*]	100 (59.0–100) [7/7]	100 (63.1–100) [8/8]	100 (75.3–100) [13/13]
	PPV, % (95% CI) [*n*/*N*]	100 (47.8–100) [5/5]	100 (2.5–100) [1/1]	100 (54.1–100) [6/6]
	NPV, % (95% CI) [*n*/*N*]	87.5 (53.9–97.7) [7/8]	100 (63.1–100) [8/8]	92.9 (67.9–98.8) [13/14]
	Accuracy, % (95% CI) [*n*/*N*]	92.3 (64.0–99.8) [12/13]	100 (66.4–100) [9/9]	95 (75.1–99.9) [19/20]

ctDNA, circulating tumor DNA; MRD, molecular residual disease; FFPE, formalin-fixed paraffin-embedded; FF, fresh-frozen; PPV, positive predictive value; NPV, negative predictive value; CI, confidence interval; *n*, number of true positive or true negative cases; *N*, total number of cases in the subgroup; *six patients in longitudinal analysis had paired FFPE-FF samples; ^#^two cases in landmark analysis had paired FFPE-FF samples.

Kaplan-Meier analysis of DFS revealed significantly worse outcomes for ctDNA-based MRD^+^ patients across all assessments. Importantly, these survival analyses are unadjusted for clinicopathological variables (e.g., stage, nodal status). Therefore, the prognostic association of ctDNA MRD status should not be interpreted as independent of these factors. Multivariable analysis was not feasible due to the limited sample size. At the landmark time point, detectable MRD was associated with shorter DFS for both FFPE-derived (*p* = 0.00011; Fig. 4a), FF-derived (*p* = 0.0047; Fig. 4b) and combined (*p* < 0.0001; Fig. 4c) analyses. Similarly, during longitudinal monitoring, ctDNA-based MRD^+^ status remained consistently associated with poorer outcomes, with highly significant associations for both FFPE-based (*p* < 0.0001; Fig. 4d), FF-based (*p* < 0.0001; Fig. 4e) and combined (*p* < 0.0001; Fig. 4f) detection. These consistent findings highlight a reproducible prognostic association of post-op ctDNA detection in this cohort, irrespective of tissue source or assessment timing, although these findings need confirmation in larger, independent studies. Risk tables summarizing the number of patients at risk at each time interval are provided below the corresponding plots.

**Figure 4:**
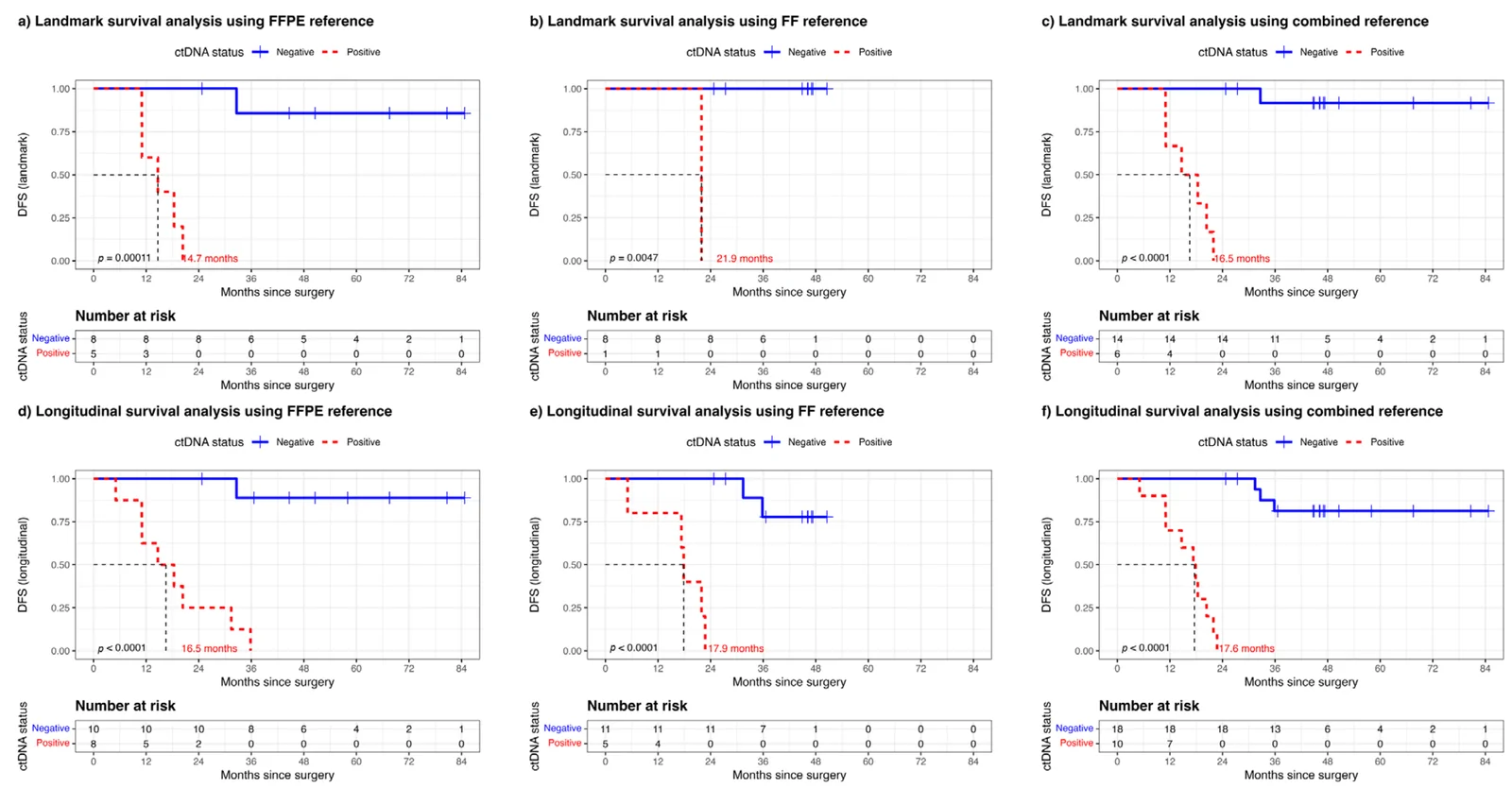
**DFS by ctDNA-based MRD status.** Landmark analysis showing significantly reduced DFS for ctDNA-based MRD^+^ patients using (**a**) FFPE, (**b**) FF, and (**c**) combined references. Longitudinal monitoring demonstrates similar prognostic value for (**d**) FFPE-derived, (**e**) FF-based, and (**f**) combined detection. The combined analysis considered a sample positive when ctDNA was detected from either FFPE-derived or FF-derived samples. ctDNA, circulating tumor DNA; DFS, disease-free survival; FF, fresh-frozen; FFPE, formalin-fixed paraffin-embedded; MRD, molecular residual disease.

Among the six matched FFPE-FF pairs (*n* = 6; statistically exploratory), MRD calls were concordant in four cases, with three concordant at the landmark time point and one during the longitudinal monitoring (Fig. 3). The two discordant cases (Patients #12 and #16) were MRD^+^ by FFPE but MRD^–^ by FF during the longitudinal monitoring. Critically, in the absence of orthogonal validation (e.g., digital droplet PCR, which could not be performed due to limited plasma volumes), it remains unresolved whether these discordant results represent a true biological signal (e.g., detection of subclones not captured by FF tissue) or residual FFPE-associated artifacts that evaded our bioinformatic filters.

### 3.5 Longitudinal Case Analysis

Longitudinal monitoring of a representative case (Patient #26) revealed distinct tumor marker dynamics (Fig. 5). Pre-op assessment showed elevated CEA and ctDNA levels, which normalized after surgical resection. During adjuvant therapy (2.6–8.2 months post-surgery), CEA levels fluctuated significantly. While ctDNA became undetectable immediately after chemotherapy completion, it later became detectable again and the cVAF continued to rise during surveillance, ultimately preceding positron emission tomography-computed tomography (PET-CT)-confirmed recurrence despite negative colonoscopy findings. The lead time for recurrence prediction using ctDNA was 7.8 months earlier than that by radiological confirmation, demonstrating the lead time advantage of ctDNA-based surveillance.

**Figure 5:**
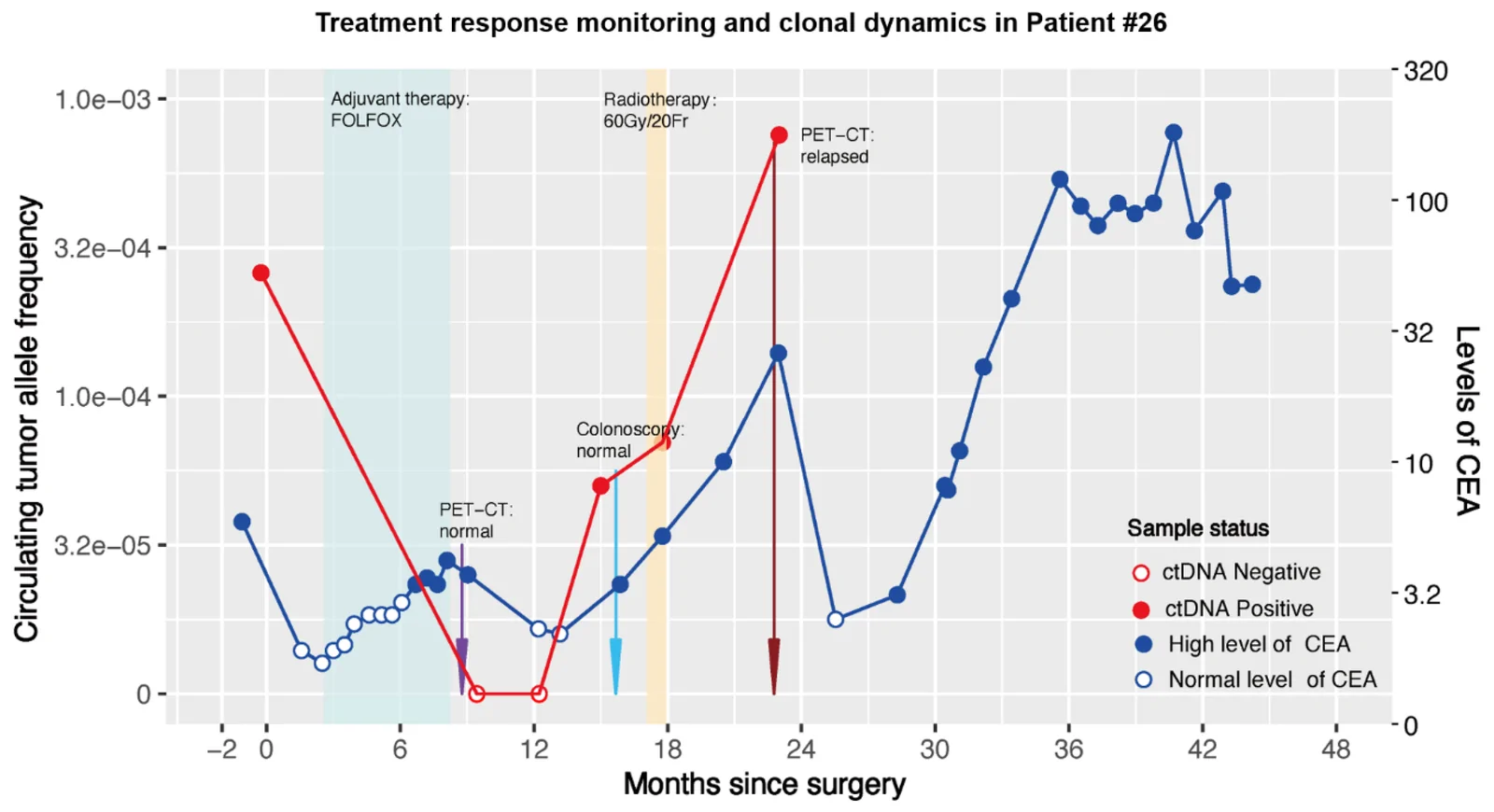
**Serial ctDNA tracking showing variant allele frequency changes preceding radiographic recurrence in Patient #26.** Arrows indicate the timing of clinical assessments of PET-CT scans and colonoscopy. ctDNA, circulating tumor DNA; PET-CT, positron emission tomography-computed tomography.

## 4 Discussion

Our study suggests that tumor-informed ctDNA analysis using the AccuScan platform provides a promising method for MRD detection in CRC patients, but these findings are hypothesis-generating given the small sample size and wide CIs. The high specificity and predictive values observed in this cohort reinforce its potential as a clinical tool for post-op monitoring and recurrence risk stratification. While we identified significant differences in mutation profiles between preservation methods, both tissue types maintained high predictive accuracy for recurrence. In a subset of patients, using both reference types together appeared to improve detection sensitivity. These findings carry important clinical implications and highlight key considerations for implementing liquid biopsies in CRC management.

In paired samples, comparative genomic profiling identified a median of 8191 somatic variants in FFPE tissue and 4261 variants in FF tissue. The significantly higher variant burden in FFPE samples is likely attributable to formalin-induced DNA damage incurred during fixation. Such damage includes fragmentation, nucleotide degradation, and protein-DNA crosslinking. A significant proportion of variants found exclusively in FFPE samples were characterized by C>T/G>A or A>G/T>C transitions. This predominance of transition mutations is consistent with established artifact patterns in FFPE-derived DNA, particularly those resulting from hydrolytic deamination. Specifically, cytosine deamination produces uracil, leading to C>T substitutions, while adenine deamination yields hypoxanthine, resulting in A>G transitions. These base alterations, exacerbated by formalin-induced DNA denaturation and suboptimal pH conditions during fixation, likely represent technical artifacts rather than true somatic mutations.

In unpaired analysis, FFPE tissues exhibited a significantly higher median variant burden than FF samples (10,277 vs. 5771 variants, *p* = 0.003), alongside lower VAFs (14.69% vs. 21.38%, *p* = 0.059). This aligns with prior reports linking FFPE processing to artifactual mutations, particularly through cytosine deamination and oxidative damage [7,12,13]. Our mutational signature analysis further supported this observation, revealing enriched A>T transversions in FFPE samples. As noted in the Results, we did not control for institutional tissue processing variables, so this finding may not be generalizable. The absence of significant C>T enrichment in our data is likely attributable to our aggressive filtering of deamination-associated artifacts. These findings suggest that ctDNA MRD detection based on FFPE samples may carry a risk of false positives due to artifacts, which can be effectively removed by appropriate bioinformatic filtering, as demonstrated in previous studies [7,12,13,18]. Unlike some previous studies [12,13], we did not observe significant C>T enrichment in our matched FFPE samples (likely reflecting the effectiveness of our bioinformatic pipeline in filtering out formalin-induced deamination artifacts). This finding suggests that with appropriate filtering, the residual mutational signal in FFPE samples aligns closely with that of FF tissue, supporting their clinical utility for tumor-informed assays. In the two discordant cases (Patients #12 and #16), the biological significance of the discordant findings remains uncertain. Potential explanations include intratumor heterogeneity, sampling-related differences, residual FFPE-associated artifacts, or false-negative results in the FF-based analysis. However, the absence of orthogonal validation precludes determination of the underlying cause. We emphasize that these cases do not provide definitive evidence for the superiority of either tissue type.

Our findings confirm and expand upon existing FFPE vs. FF molecular profiling studies. While previous reports have documented increased C>T artifacts in FFPE DNA due to cytosine deamination [7,18,19], we identified significant A>T enrichment in our FFPE samples. Basyuni et al. also reported A>T enrichment in FFPE artifacts of some samples [20]. However, given that our study included only six paired FFPE-FF samples, this difference could potentially be attributed to sample bias or artifacts. Chaudhary et al. similarly reported higher variant burdens in FFPE CRC samples, though their study did not assess ctDNA-based MRD accuracy [21]. Our work extends these observations by suggesting that FF-derived assays may also lead to false-negative MRD calls in clinical practice. The high concordance for true somatic variants between matched samples supports Fortunato et al.’s [22] conclusion that optimized experimental workflows (DNA quality control, DNA repair treatment), appropriate bioinformatics methodologies (incorporating molecular barcodes, advanced error-suppression algorithms), and FFPE-specific artifact filters [7,19,20,23] can effectively manage FFPE-related noise.

Our analysis of matched FFPE and FF samples is also subject to the confounding effects of intratumor heterogeneity. Even when derived from the same patient, these two specimens represent distinct spatial biopsies of what is often a genetically heterogeneous tumor. As a result, some variants classified as “unique” to one preservation method may actually reflect true subclonal somatic mutations present only in that particular tumor region, rather than technical artifacts.

Our results align with the growing evidence supporting ctDNA as a prognostic biomarker in CRC [6,24,25] in unadjusted analyses. Notably, the 100% specificity observed in our study is higher than some previous reports, such as the 87.9% and 86.9% specificity reported by Nakamura et al. [6,26], 90.9% reported by Parikh et al. [27], and 93% reported by Frydendahl et al. [28]. This may be due to our use of stringent variant filtering and tumor-informed assay design. In addition, the use of RCA and concatemer structures help achieve an ultra-low error rate [9,10,11], which leads to perfect specificity in MRD detection. Since our survival analyses did not adjust for other clinical or pathological factors, we cannot say that ctDNA MRD status is an independent predictor of prognosis. That question will need to be addressed with multivariable analysis in a larger group of patients. The temporal sensitivity patterns observed in our study appear to differ from the consistent sensitivity reported across timepoints in the DYNAMIC trial [5]. Given the overlapping CIs of our estimates (Table 2), this discrepancy likely reflects our modest sample size, differences in assay platforms, patient population characteristics (our cohort included more stage III-IV cases), or variations in monitoring protocols. These comparative findings highlight the importance of considering both tissue preservation methods and monitoring timelines when implementing ctDNA-based MRD in clinical practice.

The timing of ctDNA assessment is essential for obtaining reliable MRD results which requires carefully planned landmark analyses [29]. In studies investigating ctDNA-based MRD detection in CRC, the landmark time point is typically selected between 2–10 weeks after surgery [5,6,26,27,28,29]. In our study, the landmark time point of 2–6 weeks post-surgery was selected based on clinical, biological rationale, and sample availability. Clinically, this time point aligns with the National Comprehensive Cancer Network (NCCN) guidelines, which recommend that the initiation of adjuvant therapy should not exceed 8 weeks after surgery and ideally be started within 6 weeks [30]. Our landmark time point allowing assessment of baseline ctDNA-based MRD status prior to adjuvant therapy. Biologically, during the initial weeks following surgery, surgical trauma and post-op inflammation induced elevated cfDNA, which can dilute tumor-derived ctDNA, potentially resulting in ctDNA fraction below the detection limit of the assay and ultimately mask ctDNA detection in patients with residual disease [31,32].

Our findings offer several important insights for clinical practice. Notably, the widespread availability and practical advantages of FFPE tissues, including their room-temperature stability which largely facilitates their storage, handling, and transportation, seamless integration into existing diagnostic workflows, and the comparable performance metrics compared to FF samples, support the conclusion that FFPE tissues are suitable for ctDNA-based MRD assays. A key message from this work is that the assay performs robustly with FFPE tissue, which is not inferior to FF. However, to avoid false positives of MRD detection, laboratories should implement bioinformatics pipelines specifically designed to handle artifacts in FFPE-derived sequencing data. Furthermore, in cases when FFPE and FF references yield discordant MRD results, clinicians should interpret these findings cautiously and integrate complementary data (imaging, tumor markers) for comprehensive assessment. Importantly, the 100% PPV of ctDNA-based MRD^+^ results in this cohort suggests that such approaches are reliable for identifying patients with residual disease, strongly supporting its use in guiding early intervention trials, as demonstrated in ongoing studies like CIRCULATE [6]. This reinforces ctDNA’s growing role in personalized CRC management.

Our longitudinal case monitoring revealed ctDNA’s superior specificity compared to CEA for detecting recurrence. Notably, ctDNA resurgence consistently preceded radiographic relapse, even when colonoscopy results were negative. The observed pattern (undetectable ctDNA post-chemotherapy followed by delayed emergence) may represent either eradication of chemotherapy-sensitive micrometastases or expansion of resistant clones. It is also important to recognize that adjuvant therapy can transiently suppress ctDNA shedding, potentially creating false-negative MRD results during the treatment period. In Patient #26, ctDNA became undetectable immediately after chemotherapy completion but later re-emerged, illustrating this phenomenon. Consequently, sensitivity estimates from longitudinal monitoring that includes on-treatment time points may underestimate the true prevalence of residual disease. Our cohort excluded all patients receiving neoadjuvant therapy. Therefore, these findings do not generalize to the neoadjuvant setting. Given that a substantial proportion of stage III and IV CRC patients receive neoadjuvant treatment in contemporary practice, this represents a major limitation to the direct clinical applicability of our results. Preliminary clonal evolution data further underscores the value of serial ctDNA monitoring for tracking tumor dynamics. These findings suggest three practical recommendations: (1) Combine ctDNA with PET-CT when recurrence is suspected, (2) Consider ctDNA dynamics when making therapy escalation decisions, and (3) Prioritize ctDNA in surveillance protocols while maintaining CEA as supplementary. However, these clinical applications require validation through larger cohort studies to confirm their generalizability. Nevertheless, the ability to detect recurrence months before radiographic confirmation offers a critical window for early intervention and personalized treatment adjustment.

Our findings show that tumor-informed ctDNA analysis using FFPE tissue offers a robust and reliable approach for MRD detection, with performance metrics comparable to those obtained with FF tissue. This is an important consideration for clinical practice: FFPE samples are the standard of care and universally available, whereas collecting FF tissue poses logistical challenges. Our findings also highlight the practical utility of tumor-informed ctDNA assays in real-world clinical settings. The use of archival FFPE tissues, which are widely available and easily integrated into diagnostic workflows, does not compromise assay performance when appropriate bioinformatic filters are applied. This facilitates the broader adoption of ctDNA-based monitoring in routine practice.

Several limitations of our study should be noted. The modest sample size (*n* = 28), particularly the small number of matched FFPE-FF pairs (*n* = 6), limits statistical power for subgroup analyses and renders all subgroup comparisons statistically unstable and exploratory. The wide CIs throughout the results underscore the instability of these estimates, which may not be representative of broader clinical populations. The analytical framework for MRD calling was based on internal empirical observations without external validation. The absence of multivariable analysis means that ctDNA MRD status cannot be established as an independent prognostic biomarker. The retrospective design and potential selection bias from excluding patients with low cfDNA yield, who might represent tumors with low ctDNA shedding, further limit generalizability. Moreover, the limited sample size prevented us from performing a robust multivariable analysis to determine whether ctDNA MRD status is independent of traditional clinicopathological variables such as stage or nodal status. A larger study will be needed to address this important question. The exclusion of neoadjuvant-treated patients limits generalizability to contemporary CRC populations receiving pre-op therapy and the high proportion of adjuvant-treated patients (75%) may affect the generalizability of our sensitivity estimates, as therapy-induced ctDNA suppression could transiently mask residual disease. Potential selection bias exists as we excluded patients with low cfDNA yield, who may represent biologically distinct subgroups. While our dual-institution design might affect generalizability, our cohort’s clinicopathological characteristics align well with broader CRC populations. These limitations underscore the need for validation through larger, multicenter prospective studies.

## 5 Conclusion

Our findings suggest that ctDNA-based MRD detection using the AccuScan platform is feasible with both FFPE and FF reference tissues. However, due to the limited sample size, lack of external validation, and absence of neoadjuvant patients, these results require validation in larger, prospective multicenter studies before any clinical application can be recommended. We did see a significant link between ctDNA MRD status and disease recurrence in our analyses, but that is only preliminary for now. As liquid biopsy technology becomes increasingly integrated into clinical practice [26], these preliminary insights may help shape future research directions.

## Data Availability

The data that support the findings of this study are available from the corresponding author upon reasonable request.

## Supplementary Materials

The supplementary material is available online at https://www.techscience.com/doi/10.32604/or.2026.080218/s1, and includes: Table S1: Characteristics of 28 colorectal cancer patients; Table S2: Tumor-specific variant counts detected in FFPE and FF tumor samples from colorectal cancer; Table S3: Tumor-specific variant counts detected in paired FFPE and FF tissue samples; Table S4: Circulating tumor DNA detection of colorectal cancer in pre-operative and post-operative plasma samples; Table S5: The overlap of variants between pre- and post-op plasma and tumor tissue; Table S6: Cross-tables of ctDNA MRD analysis by time points and sample types. Figure S1: Distribution of mutation VAFs in matched FFPE-FF tumor pairs; Figure S2: Single-nucleotide variant distribution in matched FFPE-FF tumor pairs (*n* = 6); Figure S3: Fraction of each of the six substitution types for all tumor samples; Figure S4: Distribution of VAFs for variants selected from tissue for tracking in plasma; Figure S5: Circulating tumor DNA analysis.

## Abbreviations

The following abbreviations are used in this manuscript:

CEA	Carcinoembryonic antigen
cfDNA	Cell-free DNA
CI	Confidence interval
CRC	Colorectal cancer
ctDNA	Circulating tumor DNA
cVAF	Circulating variant allele frequency
DFS	Disease-free survival
FF	Fresh-frozen
FFPE	Formalin-fixed paraffin-embedded
gDNA	Genomic DNA
IQR	Interquartile range
MRD	Molecular residual disease
NPV	Negative predictive value
PET-CT	Positron emission tomography-computed tomography
Post-op	Post-operative
PPV	Positive predictive value
Pre-op	Pre-operative
RCA	Rolling circle amplification
VAF	Variant allele frequency
WGS	Whole genome sequencing
