# Supplementary material for: Monitoring Molecular Residual Disease in Colorectal Cancer Using Tumor-Informed ctDNA Analysis
Source: Oncol Res. 2026 Oct 28;34(11):18. doi: 10.32604/or.2026.080218 (PMC13732390; doi:10.32604/or.2026.080218)
Supplement: Supplementary file 1 [file OncolRes-34-80218-s001.zip › Figures_S1-S5.docx]

**Supplementary Figures:**

**Figure S1:** A histogram of VAF distribution for mutations unique to FFPE, mutations unique to FF, and shared mutations between both in the 6 FFPE-FF matched cases. FF, fresh-frozen; FFPE, formalin-fixed paraffin-embedded; VAF, variant allele frequency.

**Figure S2:** Single-nucleotide variant distribution in matched FFPE-FF tumor pairs (n = 6). Connecting lines show intra-patient variation between preservation methods, the Wilcoxon signed-rank test was used. FF, fresh-frozen; FFPE, formalin-fixed paraffin-embedded.

**Figure S3:** A stacked bar plot with the fraction of the six substitution types for all 28 tumor samples (FFPE and FF), with matched pairs clearly indicated. FF, fresh-frozen; FFPE, formalin-fixed paraffin-embedded.

**Figure S4:** Distribution of VAFs for the variants selected from tissue for tracking in plasma. VAF, variant allele frequency; FF, fresh-frozen; FFPE, formalin-fixed paraffin-embedded.

**Figure S5:** **Circulating tumor DNA analysis.** (**a**) Pre-op cVAF comparison between FFPE-derived (*n* = 8) and FF-derived (*n* = 13) variants. (**b**) Pre-op paired cVAF in matched FFPE-FF samples (*n* = 5) with intra-patient connections. (**c**) Pre-op cVAF distribution by tumor stage (I-II vs. III-IV). (**d**) Pre-op cVAF comparison by recurrence status. For cases with paired FFPE-FF samples, the cVAF in C-D was calculated as the mean of the cVAFs derived from each sample. Linear regression analysis of pre-op mean VAF in FFPE samples (**e**) and FF (**f**) samples with pre-op cfDNA cVAF. Linear regression analysis of pre-op cfDNA cVAF derived from FFPE samples (**g**) and FF samples (**h**) with DFS. (**i**) Post-op landmark cVAF distribution by tumor stage (I-II vs. III-IV). Box plots represent the box boundaries = IQR, with the center line = median and whiskers = 1.5 × IQR, the Wilcoxon signed rank test was used. cVAF, circulating variant allele frequency; DFS, disease-free survival; IQR, interquartile range; Post-op, post-operative; Pre-op, pre-operative; cfDNA, cell-free DNA.
