# Supplementary material for: Monitoring Molecular Residual Disease in Colorectal Cancer Using Tumor-Informed ctDNA Analysis
Source: Oncol Res. 2026 Oct 28;34(11):18. doi: 10.32604/or.2026.080218 (PMC13732390; doi:10.32604/or.2026.080218)
Supplement: Supplementary file 1 [file OncolRes-34-80218-s001.zip › Tables_S1-S6.pdf]

Table S1. Characteristics of 28 colorectal cancer patients.

Patient ID	Gender	Age	Differentiation	Tumor site	Tumor location	Stage	Tumor	Node	M	Therapy	Relapse site	Relapse	DFS (months)	Survival	OS (months)
CRC01	Female	71	Moderately	Transverse colon	Left	III	T4	N1	M0	CT	T7 Transverse colon with lap transverse colectomy	1	11.00	0	22.90
CRC02	Female	64	Well/moderately	Rectum	Left	III	T4	N2	M0	CT	Rectum	1	14.67	0	34.47
CRC03	Male	74	Moderately	Descending colon	Left	III	T3	N1	M0	CT	Colon	1	32.63	1	83.93
CRC04	Female	59	Moderately	Caecum	Right	III	T4	N1	M0	CT	Multiple: 2 Lung met + Rt anterior abdominal deposit	1	18.37	0	40.10
CRC05	Male	72	Moderately	Rectum	Left	III	T3	N2	M0	CT	Rectum with lung mets	1	11.03	0	35.60
CRC06	Male	52	Moderately	Colon	Right	III	T3	N1	M0	CT	NA	0	84.70	1	84.70
CRC07	Female	73	Moderately	Colon	Right	III	T3	N1	M0	CT	NA	0	80.67	1	80.67
CRC08	Male	59	Moderately	Sigmoid	Left	III	T2	N1	M0	CT	NA	0	67.57	1	67.57
CRC09	Male	48	Moderately	Caecum	Right	III	T4	N1	M0	CT	NA	0	67.53	1	67.53
CRC10	Male	59	Moderately	Colon	Right	III	T3	N1	M0	CT	NA	0	58.00	1	58.00
CRC11	Male	68	Moderately	Sigmoid	Left	II	T3	N0	M0	No	NA	0	47.03	1	47.03
CRC12	Female	59	Moderately	Rectum	Left	I	T2	N0	M0	No	Active interlobar nodal met	1	35.83	1	44.90
CRC13	Female	63	Moderately	Rectum	Left	II	T3	N0	M0	No	NA	0	47.33	1	47.33
CRC14	Male	74	Moderately	Sigmoid	Left	II	T3	N0	M0	No	NA	0	44.67	1	50.57
CRC15	Male	76	Moderately	Sigmoid	Left	II	T3	N0	M0	No	NA	0	46.23	1	46.23
CRC16	Female	70	Moderately	Sigmoid	Left	III	T3	N2	M0	CT	Multiple	1	31.43	1	52.40
CRC17	Female	62	Moderately	Sigmoid	Left	II	T3	N0	M0	CT	NA	0	44.90	1	44.90
CRC18	Male	72	Moderately	Rectum	Left	II	T3	N0	M0	No	NA	0	36.60	1	36.60
CRC19	Female	86	Moderately	Rectum	Left	IV	T3	N1	M1	CT	Liver only	1	21.93	1	27.53
CRC20	Male	67	Well	Transverse colon	Right	II	T3	N0	M0	No	NA	0	46.13	1	46.13
CRC21	Male	60	Moderately	Rectum	Left	III	T2	N1	M0	CT	NA	0	50.57	1	50.57
CRC22	Male	65	Moderately	Rectum	Left	III	T2	N1	M0	CT	NA	0	24.73	1	24.73
CRC23	Male	56	Moderately	Rectum	Left	III	T3	N1	M0	CT	NA	0	27.40	1	27.40
CRC24	Male	62	Moderately	Descending colon	Left	IV	T4	N1	M1	CT	Multiple: abdominal wall and lung	1	20.37	1	25.50
CRC25	Female	37	Moderately	Sigmoid	Left	IV	T3	N2	M1	CT	Peritoneal only	1	17.87	1	41.63
CRC26	Male	72	Moderately	Rectum	Left	III	T3	N1	M0	CT	Locoregional	1	22.77	1	48.33
CRC27	Female	55	Moderately	Rectum	Left	IV	T4	N1	M1	CT+Target	Lung only	1	17.33	0	24.70
CRC28	Male	59	Moderately	Rectum	Left	IV	T3	N1	M1	CT	Multiple	1	5.07	0	18.47

CT, chemotherapy; DFS, disease-free survival; NA, not applicable; OS, overall survival.

Table S2. Tumor-specific variant counts detected in FFPE and FF tumor samples from colorectal cancer patients.

Patient ID	Tissue Types	Variant counts	Mean VAF	A>C	A>G	A>T	C>A	C>G	C>T	Average Sequencing Depth
CRC01	FFPE	29896	14.10%	6.39%	11.05%	30.06%	22.78%	5.18%	24.53%	47.86
CRC02	FFPE	19167	13.41%	7.76%	12.37%	32.58%	18.34%	3.67%	25.27%	39.4
CRC03	FFPE	23112	15.28%	6.18%	10.14%	35.75%	19.03%	3.26%	25.64%	35.02
CRC04	FFPE	22649	8.96%	8.85%	15.88%	30.31%	16.04%	4.19%	24.73%	65.85
CRC05	FFPE	17135	11.52%	9.80%	16.69%	29.15%	16.91%	4.19%	23.27%	46.41
CRC06	FFPE	7087	22.28%	17.02%	17.57%	21.74%	17.75%	4.74%	21.18%	26.28
CRC07	FFPE	10596	14.07%	9.69%	15.17%	25.14%	19.53%	4.73%	25.75%	38.61
CRC08	FFPE	4491	30.63%	15.10%	17.57%	12.89%	26.21%	11.67%	16.57%	24.69
CRC09	FFPE	7094	19.74%	8.77%	14.21%	26.46%	22.39%	4.45%	23.72%	26.2
CRC10	FFPE	8139	19.73%	11.41%	15.05%	27.21%	15.42%	6.35%	24.55%	26.63
CRC11	FF	3006	32.32%	7.82%	23.82%	8.75%	26.78%	7.22%	25.62%	43.45
CRC12	FFPE	9774	26.35%	15.34%	21.09%	11.52%	23.78%	10.62%	17.66%	41.29
CRC12	FF	9052	39.50%	20.28%	21.53%	11.01%	20.80%	9.96%	16.41%	35.64
CRC13	FF	6634	18.24%	12.04%	20.64%	12.96%	25.87%	10.78%	17.71%	49.49
CRC14	FFPE	9957	25.09%	10.82%	16.50%	18.10%	22.72%	5.61%	26.25%	49.42
CRC15	FF	2031	13.53%	6.99%	20.33%	10.34%	33.28%	7.73%	21.32%	54.4
CRC16	FFPE	3559	17.19%	13.32%	19.89%	11.44%	24.75%	8.29%	22.31%	48.89
CRC16	FF	3156	16.45%	14.07%	20.25%	10.61%	24.37%	8.46%	22.24%	48.19
CRC17	FF	6630	20.74%	11.33%	19.46%	12.71%	26.38%	10.29%	19.83%	60.91
CRC18	FFPE	8763	8.53%	12.94%	17.23%	24.60%	16.03%	8.91%	20.28%	61.36
CRC18	FF	8366	18.02%	19.89%	23.28%	14.89%	15.14%	15.01%	11.77%	38.34
CRC19	FF	7035	29.86%	8.66%	18.99%	12.21%	31.43%	9.89%	18.82%	38.45
CRC20	FF	4912	22.01%	9.36%	16.25%	9.36%	35.71%	6.62%	22.70%	33.5
CRC21	FF	4029	28.51%	10.28%	20.75%	10.87%	26.68%	10.40%	21.02%	42.35
CRC21	FFPE	7618	21.78%	10.11%	18.68%	17.66%	22.46%	8.58%	22.51%	33.07
CRC22	FFPE	6829	17.88%	10.66%	18.61%	18.89%	17.16%	10.81%	23.87%	55.31
CRC22	FF	2384	23.13%	11.87%	22.19%	12.04%	19.42%	12.04%	22.44%	40.8
CRC23	FF	7508	20.35%	26.51%	25.48%	12.04%	18.59%	4.94%	12.44%	39.97
CRC24	FFPE	7326	11.39%	10.52%	16.43%	21.01%	22.44%	7.86%	21.73%	51.69
CRC25	FF	14380	30.26%	38.39%	19.31%	11.08%	14.05%	7.46%	9.69%	23.75
CRC26	FF	4900	23.34%	10.33%	21.67%	9.29%	31.12%	8.53%	19.06%	41.97
CRC27	FF	3974	18.69%	10.74%	19.75%	11.70%	27.63%	11.95%	18.22%	40.11
CRC28	FFPE	8784	21.53%	26.39%	21.84%	10.76%	21.08%	4.30%	15.63%	53.27
CRC28	FF	4492	17.10%	22.44%	21.04%	10.31%	24.96%	4.76%	16.50%	40.56

FF, fresh-frozen; FFPE, formalin-fixed paraffin-embedded.

Table S3. Tumor-specific variant counts detected in paired FFPE and FF tissue samples.

Patient ID	FFPE	FF	Overlap	Sensitivity*	Precision*	Variant counts unique in FFPE	Variant counts unique in FF
CRC28	8784	4492	4020	89.49%	45.77%	4764	472
CRC21	7618	4029	2694	66.87%	35.36%	4924	1335
CRC22	6829	2384	1868	78.36%	27.35%	4961	516
CRC16	3559	3156	2496	79.09%	70.13%	1063	660
CRC12	9774	9052	5817	64.26%	59.52%	3957	3235
CRC18	8763	8366	2390	28.57%	27.27%	6373	5976

*Performance measures of calls considering the FF sample as gold standard for variant calls.

FF, fresh-frozen; FFPE, formalin-fixed paraffin-embedded.

Table S4. Circulating tumor DNA detection of colorectal cancer pre-operative and post-operative plasma samples.

Patient ID	cVAF of Pre-op ctDNA (FFPE)	cVAF of Pre-op ctDNA (FF)	Time interval of Post-op plasma (months)	Post-op ctDNA MRD status (FFPE)	cVAF of Post-op ctDNA (FFPE)	Post-op ctDNA MRD status (FF)	cVAF of Post-op ctDNA (FF)
CRC01	NA	NA	0.90	Positive	4.67E-06	NA	NA
CRC02	NA	NA	1.17	Positive	4.45E-06	NA	NA
CRC03	NA	NA	1.10	Negative	0	NA	NA
CRC04	NA	NA	1.33	Positive	6.49E-06	NA	NA
CRC05	1.20E-04	NA	0.93	Positive	1.28E-05	NA	NA
CRC06	NA	NA	1.50	Negative	0	NA	NA
CRC07	NA	NA	1.03	Negative	0	NA	NA
CRC08	NA	NA	1.13	Negative	0	NA	NA
CRC09	NA	NA	1.27	Negative	0	NA	NA
CRC10	NA	NA	2.00	Negative	0	NA	NA
CRC11	NA	6.47E-04	0.60	NA	NA	Negative	0
CRC12	3.84E-04	2.81E-04	21.87	Positive	3.67E-06	Negative	0
CRC13	NA	NA	0.67	NA	NA	Negative	0
CRC14	1.71E-03	NA	0.47	Negative	0	NA	NA
CRC15	NA	4.43E-04	0.50	NA	NA	Negative	0
CRC16	1.54E-03	1.64E-03	15.40	Positive	9.24E-06	Negative	0
CRC17	NA	3.19E-03	1.33	NA	NA	Negative	0
CRC18	1.78E-04	3.26E-04	13.13	Negative	0	Negative	0
CRC19	NA	NA	0.73	NA	NA	Positive	4.58E-03
CRC20	NA	1.67E-03	1.33	NA	NA	Negative	0
CRC21	9.12E-04	2.11E-03	1.17	Negative	0	Negative	0
CRC22	8.20E-04	8.01E-04	0.73	Negative	0	Negative	0
CRC23	NA	1.43E-04	0.63	NA	NA	Negative	0
CRC24	7.27E-04	NA	0.60	Positive	6.19E-05	NA	NA
CRC25	NA	1.87E-03	3.53	NA	NA	Positive	8.90E-05
CRC26	NA	2.60E-04	9.43	NA	NA	Negative	0
			12.23	NA	NA	Negative	0
			15.00	NA	NA	Positive	5.38E-05
			17.77	NA	NA	Positive	6.95E-05
			23.00	NA	NA	Positive	7.56E-04
CRC27	NA	1.09E-02	5.53	NA	NA	Positive	4.78E-05
CRC28	NA	NA	2.30	Positive	3.68E-04	Positive	5.20E-04

ctDNA, circulating tumor DNA; cVAF, circulating variant allele frequency; FF, fresh-frozen; FFPE, formalin-fixed paraffin-embedded; NA, not applicable; MRD, molecular residual disease; Post-op, post-operative; Pre-op, pre-operative.

Table S5. The overlap of variants between pre- and post-op plasma and tumor tissue

Patient ID	Tumor	Variants selected from tissue for tracking	Overlap variant counts in tissue and pre-op plasma	Overlap variant counts in tissue and post-op plasma	Overlap variant counts in pre- and post-op plasma
CRC11	FF	3006	113	0	0
CRC12	FF	9052	128	1	0
CRC15	FF	2031	39	1	0
CRC16	FF	3156	197	1	0
CRC17	FF	6630	518	1	0
CRC18	FF	8366	95	0	0
CRC20	FF	4912	398	1	1
CRC21	FF	4029	422	0	0
CRC22	FF	2384		0	
CRC23	FF	7508	55	1	0
CRC25	FF	14380	868	36	7
CRC26	FF	4900	56	0	0
CRC27	FF	3974	705	12	3
CRC05	FFPE	17135	76	12	1
CRC12	FFPE	9774	192	2	1
CRC14	FFPE	9957	741	2	0
CRC16	FFPE	3559	209	2	0
CRC18	FFPE	8763	65	0	0
CRC21	FFPE	7618	399	1	0
CRC22	FFPE	6829		1	
CRC24	FFPE	7326	94	17	0

FF, fresh-frozen; FFPE, formalin-fixed paraffin-embedded; Post-op, post-operative; Pre-op, pre-operative.

Table S6. Cross-tables of ctDNA MRD analysis by time points and sample types.

Longitudinal (FFPE, n=18)	Recurrence	Non-recurrence
MRD+	8	0
MRD-	1	9

Landmark (FFPE, n=13)	Recurrence	Non-recurrence
MRD+	5	0
MRD-	1	7

Longitudinal (FF, n=16)	Recurrence	Non-recurrence
MRD+	5	0
MRD-	2	9

Landmark (FF, n=9)	Recurrence	Non-recurrence
MRD+	1	0
MRD-	0	8

Longitudinal (combined, n=28)	Recurrence	Non-recurrence
MRD+	12	0
MRD-	1	15

Landmark (combined, n=20)	Recurrence	Non-recurrence
MRD+	6	0
MRD-	1	13

ctDNA, circulating tumor DNA; FF, fresh-frozen; FFPE, formalin-fixed paraffin-embedded; MRD, molecular residual disease.
